# Reduction of photosynthetic sensitivity in response to abiotic stress in tomato is mediated by a new generation plant activator

**DOI:** 10.1186/1471-2229-13-108

**Published:** 2013-07-30

**Authors:** Jason J Wargent, Douglas A Pickup, Nigel D Paul, Michael R Roberts

**Affiliations:** 1 Institute of Agriculture and Environment, Massey University, Private Bag 11222, Palmerston North 4442, New Zealand; 2 Lancaster Environment Centre, Lancaster University, Lancaster LA1 4YQ, UK

**Keywords:** Photosynthesis, Abiotic stress, Priming, Tomato, Transcriptomics, Potassium dihydrojasmonate, Sodium benzoate, L-arginine

## Abstract

**Background:**

Yield losses as a result of abiotic stress factors present a significant challenge for the future of global food production. While breeding technologies provide potential to combat negative stress-mediated outcomes over time, interventions which act to prime plant tolerance to stress, via the use of phytohormone-based elicitors for example, could act as a valuable tool for crop protection. However, the translation of fundamental biology into functioning solution is often constrained by knowledge-gaps.

**Results:**

Photosynthetic and transcriptomic responses were characterised in young tomato (*Solanum lycopersicum* L.) seedlings in response to pre-treatment with a new plant health activator technology, ‘Alethea’, followed by a subsequent 100 mM salinity stress. Alethea is a novel proprietary technology composed of three key constituent compounds; the hitherto unexplored compound potassium dihydrojasmonate, an analogue of jasmonic acid; sodium benzoate, a carboxylic acid precursor to salicylic acid, and the α-amino acid L-arginine. Salinity treatment led to a maximal 47% reduction in net photosynthetic rate 8 d following NaCl treatment, yet in Alethea pre-treated seedlings, sensitivity to salinity stress was markedly reduced during the experimental period. Microarray analysis of leaf transcriptional responses showed that while salinity stress and Alethea individually impacted on largely non-overlapping, distinct groups of genes, Alethea pre-treatment substantially modified the response to salinity. Alethea affected the expression of genes related to biotic stress, ethylene signalling, cell wall synthesis, redox signalling and photosynthetic processes. Since Alethea had clear effects on photosynthesis/chloroplastic function at the physiological and molecular levels, we also investigated the ability of Alethea to protect various crop species against methyl viologen, a potent generator of oxidative stress in chloroplasts. Alethea pre-treatment produced dramatic reductions in visible foliar necrosis caused by methyl viologen compared with non-primed controls.

**Conclusions:**

‘Alethea’ technology mediates positive recovery of abiotic stress-induced photosynthetic and foliar loss of performance, which is accompanied by altered transcriptional responses to stress.

## Background

Plants are necessarily exposed to a variety of stresses throughout growth, many of which have a detrimental effect on growth and development. As a consequence, plants have evolved an equally wide variety of defence systems to minimise the negative impacts of stress. The development of technologies that exploit natural plant stress responses has never been of greater importance, as efforts are made to strengthen food crop provision for a growing global population in the face of current and future food supply insecurities [1,2]. Threats to plant productivity are routinely imposed by biotic stresses such as herbivory and pathogenic disease [3,4], but abiotic factors, such as temperature, drought and salinity stress, pose the greatest restriction on crop production [5]. Although new genotypes provided by both conventional breeding and genetic modification technologies offer key steps forward, practical challenges still remain regarding the uptake and provision of breeding technologies [6]. Enhancing our mechanistic understanding of plant responses to environmental stimuli in order to augment existing grower practices is therefore one important route to closing the perceived yield gap of global food crop production. Although active intervention to buffer consequential yield losses due to stress has always been an explicit component of food crop cultivation practice (e.g. the use of applied agro-chemical compounds), over time more sustainable approaches directly exploiting fundamental plant responses in crop species have been developed. These include, for example, the use of partial root-zone irrigation strategies to increase water use efficiency in crops such as maize and tomato [7,8], or the early stage exposure of leafy vegetable crops to solar ultraviolet radiation to drive enhanced photoprotection and photosynthetic productivity [9].

Equally, there is currently marked opportunity to exploit those increasingly well-defined plant signalling responses to biotic stress in order to enhance plant tolerance. For example, the exploitation of non-pathogenic rhizobacteria for induced resistance against the necrotroph *Botrytis cinerea* has been successfully demonstrated in grapevine using mutant strains of *Pseudomonas fluorescens* and *P. aeruginosa*[10]. Considering potential limitations in the application of a biotic agent to induce a desired state of enhanced plant stress protection, the use of chemical elicitors to mediate tolerance or resistance to biotic stress is increasingly receiving attention [11]. For example, it is now well established that applications of the non-protein amino acid beta-aminobutyric acid (BABA) can enhance stress responses to a variety of stimuli including drought stress and disease infection [12,13]. Despite such advances, large scale applications of ‘activator’ compounds often do not represent an economically viable option. ‘Alethea’ is a novel proprietary technology composed of three key constituent compounds; the hitherto unexplored compound potassium dihydrojasmonate (PDJ), an analogue of jasmonic acid (JA); sodium benzoate (SB), a carboxylic acid precursor to salicylic acid (SA), and the α-amino acid L-arginine (Arg) (Additional file 1). The roles of the jasmonate and salicylate groups of phytohormones have been the subject of extensive focus to date, principally with regard to cellular biosynthesis, transport and perception [14,15], and particularly, the involvement of both groups in plant defence; [16-18]. Alethea is categorised as a ‘plant health regulator’ in the alleviation of abiotic plant stress, yet has not been the focus of any published studies to date. Knowledge of the capability of technologies such as Alethea in limiting the impact of abiotic stress, and elucidation of the mechanistic nature of any induced resistance to stress, might represent a step forward in the development of plant additives which could help reduce crop losses. In order to characterise the effects of Alethea on plant biology, we focussed first on stress caused by salinity. It has been estimated that around 8% of the world’s food crop productivity could be affected by elevated Na^+^ levels [19], via an often temporally separated combination of osmotic (rapid) and ionic (acute) effects on plant growth, including reductions in stomatal aperture and net photosynthetic rate [20], in addition to longer term consequences for shoot growth [21]. The managed induction of enhanced plant tolerance to salinity has received some attention to date. For example, Jakab and colleagues [22] demonstrated reduced sensitivity to both salinity and drought stress following treatment of Arabidopsis seedlings with BABA, demonstrating an abscisic acid (ABA)-dependent response mediating protective effects. In addition, colonisation of *Populus canescens* with the ectomycorrhizal fungus *Paxillus involutus* led to increased accumulation of both ABA and SA under salinity stress [23], and previous studies have raised the possibility that JA-dependent processes may confer enhanced plant tolerance to salt-mediated effects [24].

Here, we investigated the impact of Alethea treatment in tomato plants under salinity stress. Photosynthetic and related plant gas exchange variables demonstrated a clear protective effect of Alethea, and our subsequent transcriptomics approach identified a number of genes responsive to Alethea application plus a modification of the salt stress response in the presence of Alethea. On the basis of the results, we extended our investigation to evaluate the protective effects of Alethea in response to another model photosynthetic stress, the reactive oxygen species-generating methyl viologen (paraquat). Alethea dramatically reduced the extent of necrosis in a number of key crop species following application of methyl viologen, indicating a general protection against oxidative stress by Alethea. This study provides further knowledge regarding responses to salinity stress at the transcriptome level, and confirms the potential for the use of a novel plant activator-based approach to crop protection.

## Results

### Alethea regulates photosynthetic protection against salinity stress

**Figure 1:**
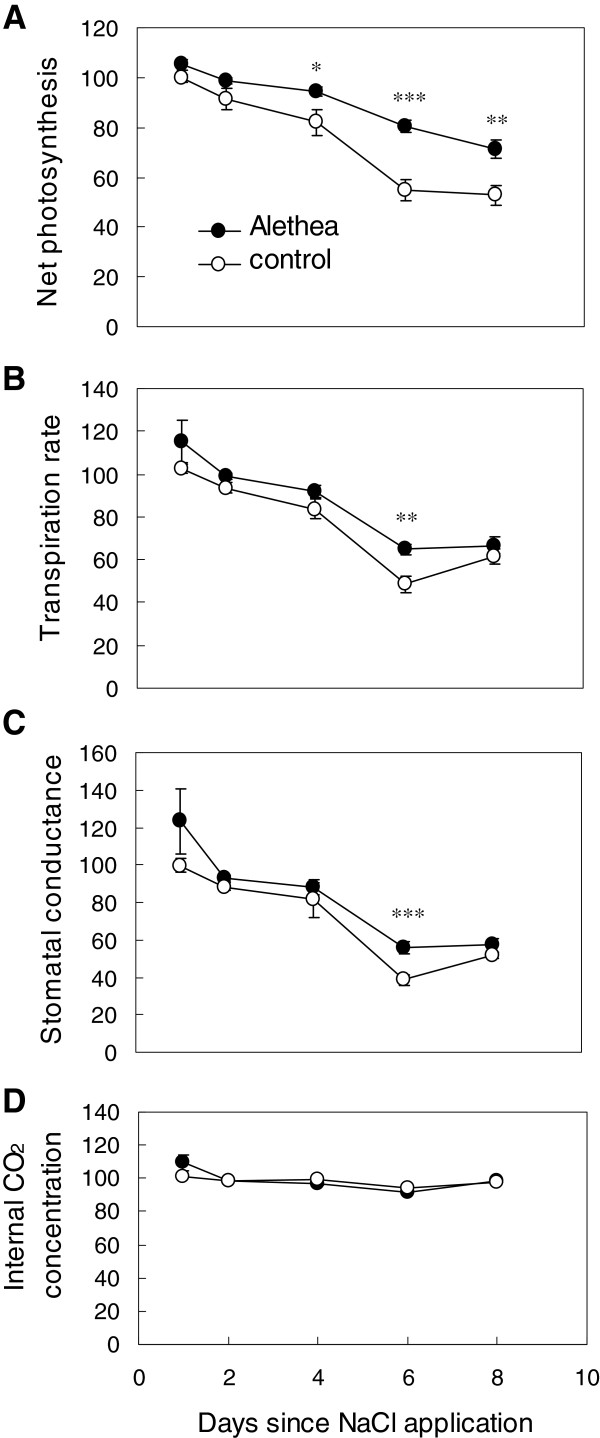
**Photosynthetic performance in primed and salt stressed tomato plants.** Gas exchange parameters as a percentage (%) of non-NaCl treated control plants were characterised in 30 day old tomato plants from application of NaCl and for eight days subsequently, following priming application of ‘Alethea’ 24 h prior to Day 0, where plants were subjected to a 100 mM salinity treatment. Both Alethea and salinity treatments were then repeated exactly as before 5 d following original treatment days (Day 4 = Alethea, Day 5 = salinity), with plants watered with H_2_O only on all other days in order to replace transpirational losses. **(A)** Net photosynthesis, **(B)** transpiration rate, **(C)** stomatal conductance, **(D)** internal CO_2_ concentration. Asterisks indicate pair-wise significant differences at * P < 0.05, ** P < 0.01, *** P < 0.001. Values are the average of 10 individual plants with standard errors.

Salinity stress is known to lead to deleterious consequences for photosynthetic performance [25]. We first characterised the protective effects of the Alethea plant activator by measuring various leaf level gas exchange parameters in young tomato plants for a period of 8 days, with Alethea and salinity stress (100 mM) applied twice in total during that period. There were no differences in photosynthetic or transpiration rates, stomatal conductance, or internal leaf CO_2_ concentration between Alethea-treated and non-Alethea treated control plants 24 h following Alethea treatment, i.e., immediately prior to application of salinity stress (Net photosynthesis: Alethea = 16.28 ± 0.27 μmol CO_2_ m^-2^ s^-1^, H_2_O = 15.91 ± 0.30 μmol CO_2_ m^-2^ s^-1^; *P* > 0.05). Following salt treatment, salinity stress caused a marked reduction in various gas exchange variables, notably in non-Alethea treated control plants, where overall photosynthetic rate was significantly reduced by salinity across the measurement period (*P* < 0.001; Figure 1A), and on a time-point basis, photosynthetic rate had decreased by 47.3% by Day 8 as compared to Day 0 values (*P* < 0.001; Figure 1A). Similarly, transpiration rate was decreased in response to salt treatment over the whole measurement period (*P* < 0.001; Figure 1B), and was significantly reduced by 38.5% by Day 8 (*P* < 0.001; Figure 1B). Stomatal conductance was significantly lower in response to salinity stress across the measurement period (*P* < 0.001; Figure 1C), and was significantly decreased by 48.3% at Day 8 (*P* < 0.001; Figure 1C). In Alethea treated plants, overall photosynthetic rate was still somewhat reduced in response to salt (*P* < 0.05; Figure 1A), but was significantly higher than non-primed salt-treated control plants across the entire measurement period (*P* < 0.001, Figure 1A), with significant increases in photosynthetic rate compared to non-Alethea salt treated plants observed on Days 4, 6, and 8 of 12.3, 25.5, and 18.4% respectively (*P* < 0.05, *P* < 0.001, *P* < 0.01; Figure 1A). Transpiration rate was significantly higher in Alethea-primed salt treated plants across the whole measurement period compared to controls (*P* < 0.001; Figure 1B), and particularly on Day 6, where Alethea-treated plants exhibited a 16.2% increase in transpiration rate as compared to salinity-stressed control plants (*P* < 0.01; Figure 1B). Similarly, stomatal conductance was significantly elevated in Alethea-treated plants across the measurement period (*P* < 0.001; Figure 1C), with a 17.4% increase in stomatal conductance rate by Day 6 as compared to non-Alethea treated controls (*P* < 0.001; Figure 1C). Internal CO_2_ concentration did not alter according to Alethea treatment in response to salinity stress during the measurement period (*P* > 0.05; Figure 1D). In summary, treatment of tomato plants with Alethea leads to a marked increase in photosynthetic tolerance towards subsequent salinity stress.

### Alethea drives transcriptional reprogramming and modifies the response to salinity

In order to examine the impact of Alethea on the plant response to salt at the molecular level and to develop an understanding of the mechanisms underlying its protective effect on leaf physiology, we performed a transcriptomic analysis using Affymetrix Gene Chip technology. Plants were pre-treated with water or Alethea, and then subjected to control (water) or salt treatments 24 h later. After a further 24 h, leaf tissues were harvested for microarray analysis. Genes responding to either Alethea or salinity treatment were identified from the appropriate pair-wise comparisons using Rank Product analysis [26] with a 5% false discovery rate used to define differentially-regulated probe sets. This approach revealed a total of 223 probe sets responsive to salt and 388 that were regulated by Alethea treatment. There were 51 probe sets common to both responses. The full list of differentially-regulated genes is provided in Additional file 2. Amongst the genes differentially-regulated by salinity, we could identify a substantial number that had previously been identified as salt responsive in tomato [27], including several transcription factors, cell wall proteins and cell wall-modifying enzymes and various stress-related genes. One of the largest changes in expression we observed was the down-regulation of proline oxidase, a well known response to drought and osmotic stress consistent with an accumulation of proline which acts as a compatible osmolyte [28]. Analysis of the differentially-expressed gene sets using gene ontology (GO) classifications identified several biological processes that appear to be regulated by Alethea or salinity (Table 1). GO terms over-represented amongst genes up-regulated by salinity treatment include protease inhibitors, ethylene receptors and genes involved in amino acid catabolism and negative regulation of ABA signalling, whilst genes involved in cell wall organisation, and in particular xyloglucan endotransglycosylases (XETs), were down-regulated. For Alethea treatment, “response to biotic stimulus” , “defense response” and “response to stress” were over-represented amongst up-regulated genes. However, within those groups, we did not identify significant numbers of genes typically associated with either JA or SA signalling pathways, such as JA or SA biosynthesis genes, or classic markers for JA-responses in tomato such as proteinase inhibitors, polyphenol oxidase, leucine amino peptidase, threonine deaminase, or SA markers such as PR genes.

**Table 1:** Enriched GO terms from the tomato function, process and component ontologies with P-value < = 0.05 (with permutation correction) for genes up- or down-regulated by salt or Alethea treatment

**Expression class**	**Ontology category**	**Gene Ontology term**	**Corrected P-value**
Salt: up-regulated	Function	Protease inhibitor activity	<0.001
	Function	Two-component sensor activity	0.005
	Function	Acetylornithine deacetylase activity	0.037
	Process	Response to stimulus	0.013
	Process	Amino acid catabolic process	0.014
	Process	Peptidyl-histidine phosphorylation	0.024
	Process	Negative regulation of abscisic acid mediated signaling	0.025
	Process	Nitrogen compound catabolic process	0.027
Salt: down-regulated	Function	Xyloglucan:xyloglucosyl transferase activity	<0.001
	Function	Structural constituent of cell wall	0.037
	Function	Delta12-fatty acid dehydrogenase activity	0.037
	Function	Omega-6 fatty acid desaturase activity	0.037
	Process	Glucan metabolic process	<0.001
	Process	Cell wall organization	0.002
	Process	Cellular carbohydrate metabolic process	0.023
	Component	Extracellular space	<0.001
	Component	Cell wall	<0.001
	Component	Apoplast	0.002
Alethea: up-regulated	Function	Acetyl-coA C-acyltransferase activity	0.022
	Function	Flavonoid 3’,5’-hydroxylase activity	0.028
	Process	Response to biotic stimulus	0.015
	Process	Defense response	0.016
	Process	Response to stress	0.036

**Figure 2:**
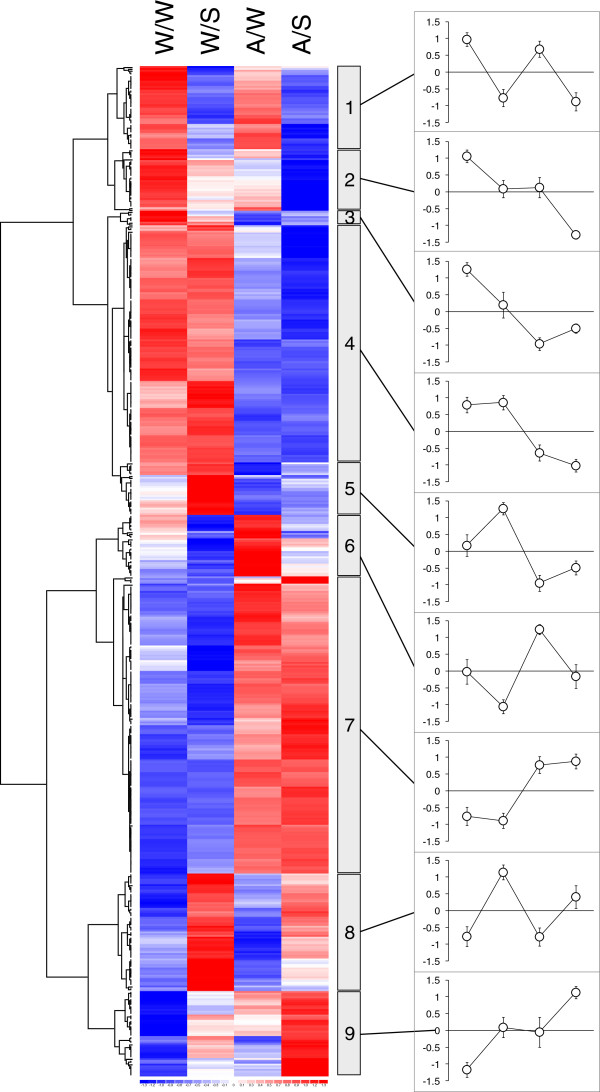
**Hierarchical clustering of differentially-regulated genes.** Heatmap display of the clustered non-redundant list of differentially-regulated probe sets derived from Rank Products analysis. Colour scheme represents standardised expression signals relative to the mean (white) for each probe set, with increasing red intensity representing high levels of expression, and blue, low expression relative to the mean. Clusters of genes showing different patterns of regulation are identified at the right of the cluster diagram, and the respective trends in expression for each cluster are illustrated by mean expression profiles for standardised data (± standard deviation) for all genes within each cluster. Notation designates Pre-treatment/Treatment, where W = water, A = Alethea, S = salt.

**Figure 3:**
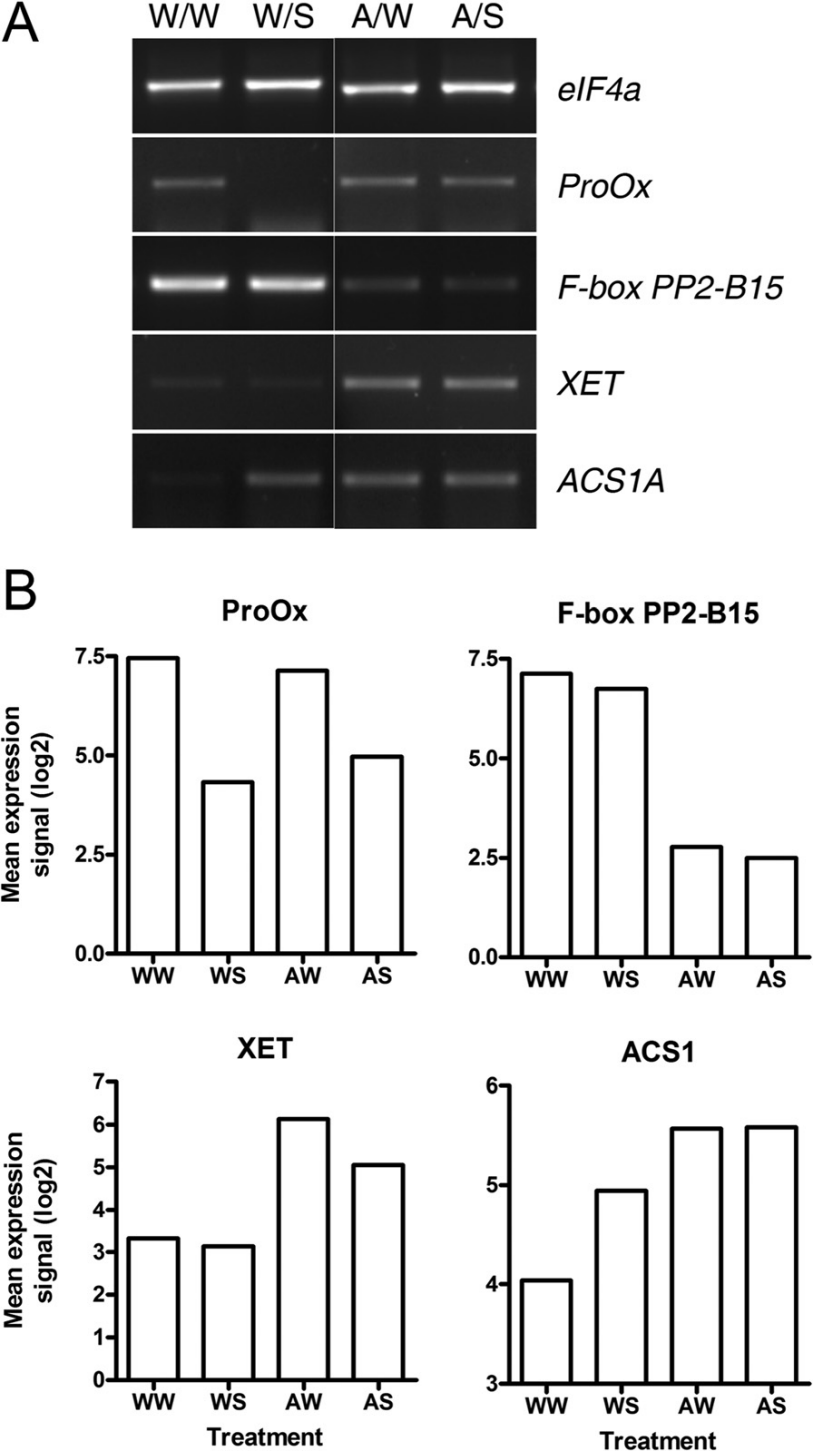
**RT-PCR assays confirm expression of differentially-regulated genes. (A)** RT-PCR products generated using cDNA templates from RNA extracted from one of the microarray replicate experiments. *ProOx*; proline oxidase (Unigene Les.1610), *F-box PP2-B15* (Unigene Les.23296), *XET*; xyloglycan endo-transglycosylase (Unigene Les.429), *ACS1A*; 1-aminocyclopropane-1-carboxylate synthase 1A (Unigene Les.1841). *eIF4a*; eukaryotic translation initiation factor 4a (Unigene Les.5856), is included as an internal loading control. **(B)** Plots of mean Affymetrix log_2_ expression signals from the three replicate microarray experiments for the corresponding genes shown in **(A)**. Notation designates Pre-treatment/Treatment, where W = water, A = Alethea, S = salt.

Hierarchical clustering was performed to visualise the patterns of regulation of the differentially-expressed genes. By visual inspection of the resulting cluster diagram (Figure 2), we identified nine gene clusters representing distinct combinations of responses to Alethea and salinity (a full list of annotated genes and associated GO terms for each cluster are available in Additional file 3). The largest two clusters (Clusters 4 and 7) represent genes which respond to Alethea treatment independently of salinity stress. Clusters 1 and 8 meanwhile, contain genes that are regulated by salinity. These responses are largely independent of Alethea, although there is some evidence of an attenuation of the magnitude expression of salt-induced genes by Alethea pre-treatment. Of the remaining clusters, clusters 2,6 and 9 contain genes that show additive responses to salt and Alethea. Two clusters, however, represent more complex interactions between salt and Alethea. Cluster 5 contains genes that are up-regulated by salinity in control plants, but not in plants pre-treated with Alethea, whilst cluster 3 contains genes down-regulated by salt only in control plants. Alethea therefore appears to attenuate the salt-responsiveness of these groups of genes. To validate the microarray data, we selected representative genes regulated by Alethea and/or salinity for analysis by reverse transcription (RT) PCR. The results (Figure 3) generally show close agreement with the array data.

**Figure 4:**
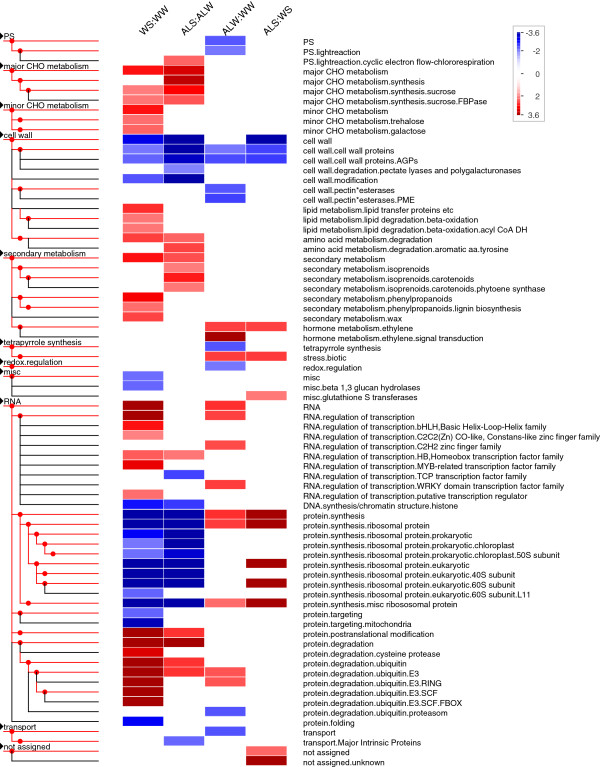
**PageMan display of MapMan gene categories affected by Alethea and salinity.** The Wilcoxon rank sum test was used to identify functional groups where the distribution of responses within a group differed from the response of the entire gene set under test. The figure is based on gene expression ratio data for relevant pair-wise treatment comparisons. Treatment groups are identified using two-letter abbreviations, where the first letter indicates the pre-treatment (W; water, A; Alethea) and the second letter the main treatment (W; water, S; salt). Coloured boxes indicate statistically-significant groups (Benjamini & Hochberg-corrected P-value below 0.05). The colour scale represents z-transformed P-values, with red shades indicating a trend within the group for up-regulation of expression relative to the control, and blue shades, down-regulation. Text alongside each row provides MapMan annotation of differentially regulated gene classes.

**Figure 5:**
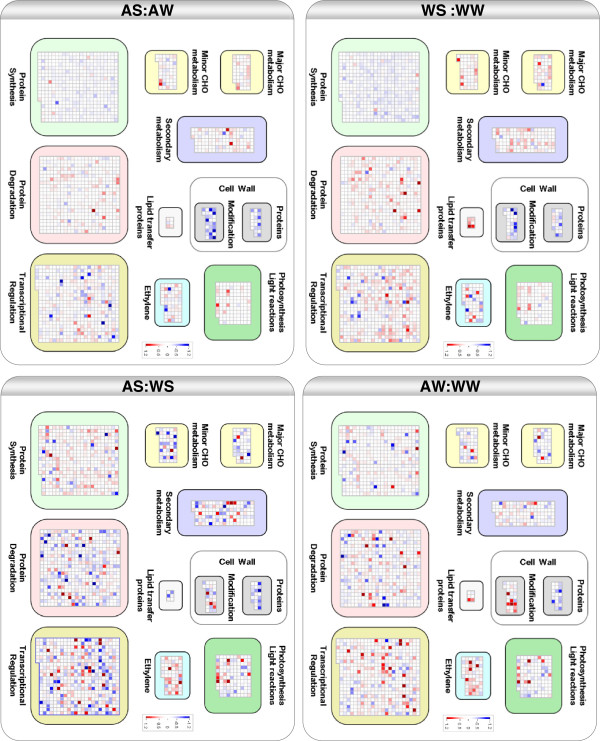
**Summary of biological pathways affected at the transcriptional level by Alethea and salinity.** Custom MapMan diagram showing changes in gene expression in key biological processes significantly altered by salt and Alethea treatments. The figure is based on gene expression ratio data for relevant pair-wise treatment comparisons. Treatment groups are identified using two-letter abbreviations, where the first letter indicates the pre-treatment (W; water, A; Alethea) and the second letter the main treatment (W; water, S; salt). Squares show changes in expression of individual genes within a pathway via a heat map scheme, with red indicating up-regulation and blue, down-regulation of expression. Heat map scales to the right of each image indicate log2 fold-change expression values.

To gain a better understanding of the biological processes affected by salinity and Alethea, we used MapMan software [29] to display the microarray data on biological pathway maps. Figure 4 presents a visual summary of those groups that were significantly affected under at least one treatment condition, and shows that salinity and Alethea affect distinct collections of biological processes. The full statistical results are provided in Additional file 4. We produced a custom MapMan diagram containing the main differentially-regulated processes to allow easy visual comparisons between treatment effects (Figure 5). Figures 4 and 5 show that Alethea treatment caused significant effects in several areas of the transcriptome. Genes showing up-regulation include those in processes related to biotic stress, ethylene signalling, some transcription factor families and some areas of protein synthesis. Processes in which genes tended to be down-regulated by Alethea include cell wall structure, tetrapyrrole synthesis and redox regulation (particularly glutaredoxins). Interestingly, genes encoding proteins with roles in the light reactions of photosynthesis were also significantly altered by Alethea.

MapMan revealed significant effects of salinity over a wider range of processes. In the absence of Alethea, the most significant effect was on protein turnover, with protein synthesis being down-regulated along with a concomitant up-regulation of genes involved in protein degradation. Several classes of transcriptional regulators were also induced, whilst arabinogalactan proteins (AGPs) and cell wall-modifying enzymes were down-regulated. In Alethea-treated plants, the overall response to salinity was broadly similar. However, the responses of several categories of genes appeared to be attenuated, whereas some responses were enhanced. For example, Alethea pre-treatment reduced salt-induced changes in genes involved in protein turnover, whilst changes in cell wall genes were enhanced. Furthermore, some classes of genes appeared to be significantly altered by salinity only in control plants or only following Alethea pre-treatment. For example, salt-induced changes in genes related to lipid metabolism, lignin and wax metabolism, and various transcription factor families were significant only in the absence of Alethea, whereas carotenoid metabolic genes were induced only in Alethea-treated plants. Overall, the main impression is that salinity and Alethea affect different but overlapping patterns of gene expression, and that the salt stress response is substantially reduced following Alethea pre-treatment.

### Protection of photosynthetic capability mediated by Alethea is wide-ranging

**Figure 6:**
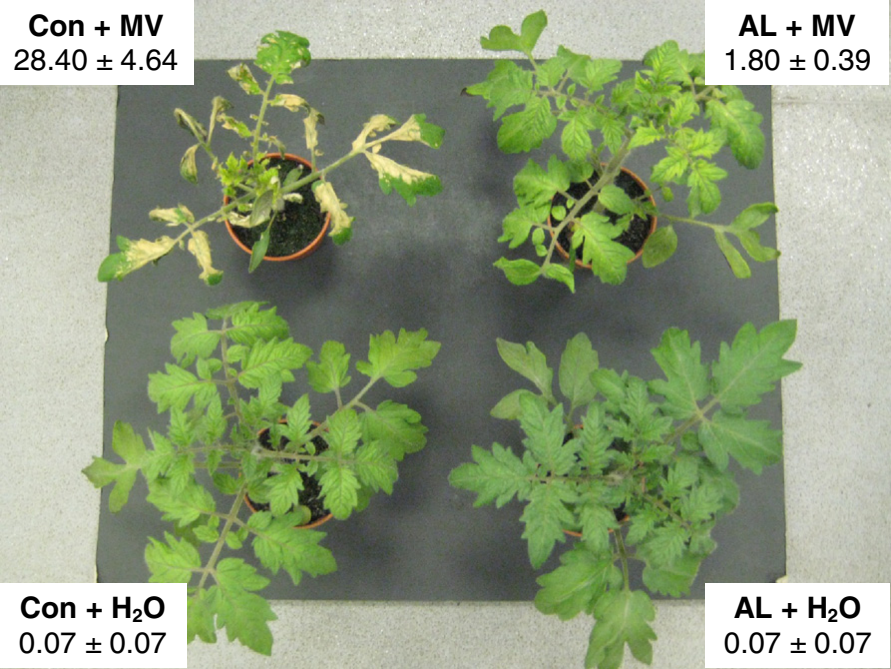
**Alethea protects plants against the photosynthetic inhibitor, methyl viologen.** Images of representative plants treated with either a water control, or 500 μM MV 24 h following water (Con) or Alethea (AL) pre-treatment. Figures adjacent to each plant show mean % visual damage +/- S.E., assessed as necrosis of plant foliage 3 d after MV treatment. Con + MV treated plants exhibit significantly higher % visual damage than all other treatments according to ANOVA (*P* < 0.001), and means presented are averaged from four individual experiments consisting of a minimum of five plants per treatment on each occasion (ANOVA for inter-experimental difference = *P* > 0.05).

The transcript analysis suggested that Alethea both modifies the salt stress response and adds an additional level of modification of gene expression, including effects on redox regulation and photosynthetic gene expression. Further inspection of the photosynthetic genes up-tregulated by Alethea in Mapman revealed four NAD(P)H dehydrogenase (NDH) subunits. The NDH complex has been implicated in photosynthetic tolerance to various abiotic stresses, particularly under conditions that decrease CO_2_ assimilation [30-32]. We therefore reasoned that the protective effect of Alethea on photosynthetic performance under salt salinity may extend to other abiotic stresses. To test this hypothesis, we treated tomato, maize, wheat, brassica and bean plants with methyl viologen (‘MV’; also known as *N*,*N*′-dimethyl-4,4′-bipyridinium dichloride or Paraquat), a herbicide which is reduced by Photosystem I to generate reactive oxygen species in chloroplasts [33]. Application of MV to control plants caused high levels of visible necrosis as a result of oxidative damage, whereas symptoms in plants pre-treated with Alethea were dramatically reduced (Figure 6; Table 2).

**Table 2:** Alethea provides protection to the photosynthetic apparatus of a range of cultivated species

	**Foliar damage (% necrosis)**			
**Treatment**	***Z. mays***	***T. aestivum***	***B. napus***	***P. vulgaris***
Con + H_2_O	0.42 ± 0.15^a^	0.00 ± 0.00^a^	0.08 ± 0.07^a^	0.17 ± 0.11^a^
AL + H_2_O	0.58 ± 0.15^a^	0.00 ± 0.00^a^	0.00 ± 0.00^a^	0.42 ± 0.15^a^
Con + MV	62.08 ± 6.38^c^	75.00 ± 3.20^c^	70.33 ± 7.90^c^	74.75 ± 3.95^b^
AL + MV	23.75 ± 2.05^b^	12.33 ± 1.79^b^	34.00 ± 4.16^b^	4.50 ± 0.81^a^
n	12 (2)	12 (2)	14 (2)	12 (2)

Values indicate visually assessed necrosis as a percentage of foliar canopy cover in four species, 3 d following treatment with either a water control, or 500 μM methyl viologen (MV) 24 h after a water (Con) or Alethea (AL) pre-treatment, ± S.E. Letters indicate significant differences between treatments according to one-way ANOVA and Tukey (*P* < 0.05), and lower row indicates number of plants assessed in total, with number of replicate experiments in brackets; replicate plants were pooled following ANOVA confirmation of non-significance between replicate experiments.

## Discussion

**Figure 7:**
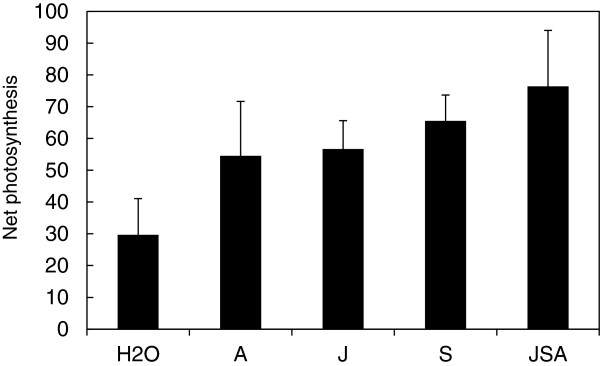
**Effects of Alethea components upon photosynthetic performance in salinity-stressed tomato plants.** Values show net photosynthesis in salt-stressed tomato seedlings as a percentage of non-salt treated plants 8 d following salinity treatment (100 mM), with a pre-treatment of Alethea components 24 h prior to salt application. Potassium dihydrojasmonate (‘J’), sodium benzoate (‘S’), and the α-amino acid L-arginine (‘A’) were applied at the same concentrations as found in the Alethea compound and in exactly the same manner as all other experiments, either singly, or in combination with all other components (‘JSA’). Means shown are of 5 replicate plants per treatment ± 1 S.E.

The commercial plant activator product ‘Alethea’ contains a combination of agents designed to promote plant stress tolerance based on changes in the activities of a range of metabolic and signalling pathways. Here, we demonstrate protective effects of Alethea on photosynthesis in plants under salinity stress and identify underlying transcriptional reprogramming events which may underpin such physiological changes. Alethea includes low concentrations of jasmonate and salicylate derivatives along with the amino acid, arginine. There is some evidence that salicylates can mediate protection against various aspects of abiotic stress, including salinity [34]; for example, consistent with the data presented here, Stevens *et al.*[35] showed that application of a root drench containing 0.1 mM SA significantly reduced the impact of salinity on photosynthesis, transpiration and stomatal conductance in tomato. Similarly, the alleviation of salt stress (including effects on photosynthesis) by pre-treatment of plants with jasmonates has been demonstrated in pea and barley [36-38]. In tomato, JA is required for salt-induced gene expression [39] and activation of JA signalling can promote salt tolerance [40]. However, in comparison with the majority of reports dealing with the exogenous application of salicylates and jasmonates, their concentrations in Alethea are rather low. For example, the majority of studies focused on jasmonate responses in tomato have tended to apply JA concentrations in the range of 1.0-1.5 mM [41-43], and while there is a good deal of variation in the SB/SA literature, concentrations in the range of 0.5-1.0 mM are not uncommon [44-46]. Whilst JA and SA can act synergistically when applied together at lower concentrations [47], our microarray analysis did not reveal patterns of gene expression typical of either JA or SA-mediated effects following Alethea treatment. For example, whilst application of methyl jasmonate to leaves of tomato plants resulted in the up-regulation of genes in the JA biosynthetic pathway and classic markers of JA responses such as proteinase inhibitors and polyphenol oxidase [48], we did not observe similar effects in Alethea treated plants. Nor did we observe widespread expression of SA-responsive PR genes. Moreover, following a small-scale screen of salinity response in tomato plants following treatment with individual or combined components of the Alethea compound (Figure 7), no clear synergistic response of combining PDJ, SB and Arg compounds was evident, and there was no significant difference between the differing treatments in this element of our study. At the same time, the current study has not explored all possible interactions between the three components of Alethea. It remains unclear whether the effect of Alethea is a simple combination of the effects of the individual constituents, or a more complex interaction between them.

Nevertheless, our microarray analysis identifies possible mechanisms underlying the protective effect of Alethea. Firstly, it is clear that rather than acting simply to enhance the existing transcriptional response to salinity, Alethea treatment generated significant changes in transcription prior to the application of salt stress, and some of these may impact on the subsequent ability of the plant to tolerate salinity. Notably, MapMan and GO term enrichment analysis both identified various defence/stress associated processes as being regulated by Alethea, which may contribute to increased tolerance. These include up-regulation of ethylene signalling and stress-associated transcription factors. Ethylene is important in a range of responses to abiotic stress, including salinity [49]. Secondly, it is also clear from the microarray data that the response to salinity is substantially affected by Alethea pre-treatment. For a number of individual genes (Figure 2; clusters 3, 5 and 8), the response to salinity is attenuated by Alethea pre-treatment, whereas for other genes (Figure 2; clusters 2 and 9), there are additive affects of salinity and Alethea which could contribute to enhanced stress tolerance. At the biological process level, as revealed by MapMan analysis, Alethea appears to augment the overall response of cell wall proteins to salt, with a number of arabinogalactan proteins (AGPs), xyloglucan endotransglycosylases (XETs) and expansins being down-regulated, consistent with a reduction in cell expansion and growth. Curiously though, Alethea treatment alone up-regulates expression of several XETs and expansins (Figure 5; cell wall modification). Previous studies have also highlighted the importance of modifications to cell wall structure in the response to salinity [50,51] and cell wall-related genes were strongly regulated by salt stress in tomato roots [27]. Since some of these classes of cell-wall genes were already altered by Alethea pre-treatment, this along with the enhanced affect upon subsequent salinity treatment may contribute to enhanced tolerance.

One of the key impacts of abiotic stress in plants is oxidative stress, resulting from over-reduction of the photosynthetic electron transport system by reduced CO_2_ availability associated with stomatal closure. Stomatal conductance was reduced by salinity in both control and Alethea-treated plants in our experiments (Figure 1). Under such conditions, photo-oxidative stress is minimised by the utilisation of alternative electron transport systems in chloroplasts. One such mechanism is the reduction of NAD(P)H by the plastidial NDH complex [30-32]. Statistical analysis in MapMan revealed that genes of the light reactions of photosynthesis were significantly affected by Alethea, and close inspection of these genes revealed four NDH subunit genes that were up-regulated by Alethea. NDH activity is increased under a range of stress conditions [30,32] and Horváth et al., [31] found that a loss-of-function NDH mutation in tobacco caused increased photosynthetic depression when CO_2_ supply was limited by stomatal closure. Hence, increased NDH expression following Alethea treatment may contribute to the protection of photosynthesis upon subsequent salt stress.

**Figure 8:**
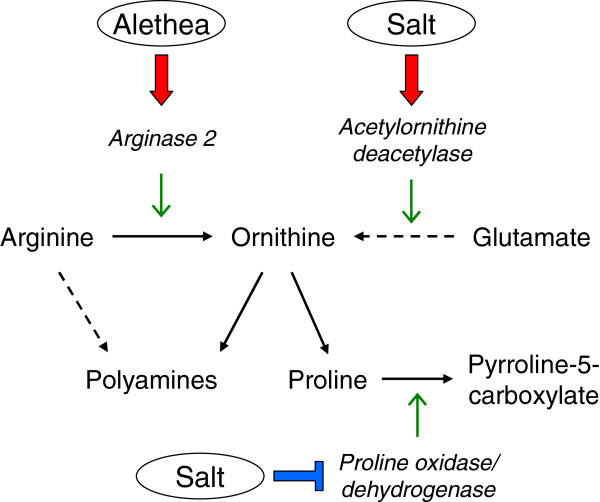
**Effects of Alethea and salinity on proline and polyamine biosynthesis.** Pathway diagram illustrating conversion of arginine and glutamate to ornithine and thence proline. Significant transcriptional effects of Alethea and salinity on genes encoding relevant enzymes (italics) are shown as red arrows for up-regulation and blue bars for down-regulation.

This mechanism would be expected to provide protection of photosynthesis under a range of abiotic stresses. This was confirmed using methyl viologen to generate chloroplast oxidative stress (Figure 6). Consistent with this, the ability of SA and JA to protect plants against salinity and other abiotic stresses has been suggested to result at least in part, from an up-regulation of antioxidative biochemistry [34,52-54]. Arginine (Arg), the third active ingredient in Alethea, has also been shown to activate antioxidant enzyme activity in tomato fruit, and exogenous application of Arg provided protection against chilling stress [55]. One explanation for the effect of Arg on antioxidant activity is its role in polyamine metabolism, where both Arg and its derivative, ornithine, are substrates for polyamine synthesis (Figure 8) [56]. Polyamines have been proposed to play important roles in abiotic stress tolerance, including via direct and indirect effects on antioxidants [57]. Ornithine also acts as a precursor for proline biosynthesis, which is also a component of drought and salinity tolerance. Our microarray analysis identified several changes in gene expression which point to a role for Alethea in the up-regulation of proline and perhaps also polyamine biosynthesis. Genes encoding arginase and acetylornithine deacetylase, enzymes for ornithine biosynthesis, were up-regulated by Alethea and salt respectively, implying increased ornithine biosynthesis, whilst proline oxidase was strongly down-regulated by salinity, consistent with an increase in proline accumulation; these events are summarised in Figure 8. Moreover, arginase gene expression has previously been shown to be responsive to both application of exogenous Arg and JA [54,56].

## Conclusion

While there is a growing body of evidence to indicate the dynamic and complex nature of plant phytohormone interactions *in planta*, fewer studies to date have provided a feasible application for incorporation of novel fundamental knowledge regarding plant activation for stress tolerance into a likely agronomic solution. Although the ‘Alethea’ technology is composed of several biologically active constituents, the end result mediates positive recovery of abiotic stress-induced photosynthetic and foliar loss of performance, based on an additive and complementary breadth of responses at the transcriptome level. Building fundamental understanding of the interactions between component compounds will be a valuable future step forward, and will further empower the buffering of food crop cultivation against intolerable losses in yield.

## Methods

### Plant propagation and growing conditions

Tomato seed (cv. Ailsa Craig, Moles Seeds Ltd, Colchester, UK) were sown and germinated in Levington M3 compost (Henry Alty Ltd., Preston, UK), prior to individual transplantation into 2 L pots. Seedlings were maintained in glasshouse conditions supplemented with high pressure sodium lighting, supplying a background Photosynthetically Active Radiation (PAR) photon flux density of approximately 500 μmol m^-2^ s^-1^, with a photoperiod of 14 h/10 h light/dark, and air temperatures of 22°C/18°C day/night. Plants were arranged randomly according to treatment, and were grown for four weeks prior to establishment of experimental treatments (approximately 6^th^ true leaf stage). For the methyl viologen assays, tomato was propagated as above; wheat (*Triticum aestivum* L.cv Granary, Quantil Ltd., Lancashire, UK), dwarf French bean (*Phaseolus vulgaris* cv. Nassau, Moles Seeds Ltd. Essex, UK) and maize (*Zea mays* cv. F1 Earligold, Moles Seeds Ltd. Essex, UK) seed were pre-germinated in dishes lined with paper towel which had been soaked in water, covered and then placed into the glasshouse under the same conditions as described above. After three days viable seeds were then selected and sown into individual pots. Bean seeds were sown into standard 13 x 14 cm pots whereas the maize and wheat were sown into 11 x 13.5 cm pots all using Levington M3 compost. For *Brassica napus* (L. cv Expert, Limagrain Ltd., Lincolnshire, UK) several seeds were sown into standard 13 cm pots as above, with seedlings thinned to single plants in each pot following emergence. Wheat, bean, maize and brassica plants were grown for 3.5 weeks before receiving any treatment.

### Pre-treatment of tomato plants with Alethea compound and salinity stress

An experimental formula of the ‘Alethea’ technology (Plant Impact PLC, Harpenden, UK) was applied to four-week old tomato seedlings at a concentration of 99:1 v/v (distilled H_2_O:Alethea) as per manufacturer’s instructions (see Additional file 1 for a detailed description of the Alethea formulation), in addition to an equal quantity of control plants, which were sprayed with distilled H_2_O. Alethea solution was sprayed onto leaves until run-off using a pressurized airbrush, plants were air-dried and then returned to the glasshouse. 24 h following Alethea application, a salinity treatment of 100 mM NaCl (Sigma-Aldrich Ltd., Dorset, UK) was applied to plants via pot-watering until maximum soil saturation was reached (~ 3 h), with control plants fed with H_2_O only. Both Alethea and salinity treatments were then repeated exactly as before 5 d following original treatment days (Day 4 = Alethea, Day 5 = salinity), with plants watered with H_2_O only on all other days in order to replace transpirational losses. For the methyl viologen assays, due to the waxy composition of the brassica leaves and vertical structure of the maize leaves, a wetting agent (Silwet L-77; De Sangosse Ltd., Cambridge, UK), was added (0.025% concentration) to the Alethea solution (and water control) when being applied to the plants, allowing the treatment to be applied evenly across the whole plant.

### Measurement of gas exchange parameters

Net photosynthesis and related gas exchange variables were measured using a portable infrared gas analysis system (CIRAS-2; PP systems, Hitchin, UK) with cuvette conditions set to PAR: 500 mmol m^-2^ s^-1^, 60% relative humidity and 380 ppm CO_2_, with leaves left to equilibrate for 5 min prior to measurement. Measurements were made using 3^rd^ true leaves and were taken daily prior to and during initial Alethea and salinity treatments, and every 48 h thereafter. Ten plants were measured per treatment in a single experiment.

### Transcriptomics experiments

24 h following salinity treatment (48 h following Alethea treatment), 3^rd^ true leaves of plants were snap frozen in liquid N_2_. Leaves were sampled from three plants per treatment, and the experiment was carried out on three separate occasions. RNA was then extracted using a scaled-up version of the method described by [58] and purified using the Qiagen RNeasy kit, as per manufacturers’ instructions (Qiagen; http://www.qiagen.com*)*. Labelling and hybridization to the Affymetrix GeneChip^®^ Tomato Genome Array were performed at the Nottingham Arabidopsis Stock Centre (University of Nottingham, UK; http://www.arabidopsis.info).

### Microarray data analysis and bioinformatics

Raw data were normalised using GCRMA [59] and the data were filtered to eliminate probe sets for which the mean signal from the three replicate arrays did not exceed a value of 10 (log2 = 3.2) for at least one treatment. This resulted in the inclusion of 7,799 probe sets for further analysis from the original 10,209 probe sets on the array. Differentially-expressed genes were identified using the Rank Product algorithm [26] implemented in the Multiple Experiment Viewer package [60]. We used 2-class paired comparisons with *P*-values calculated using 1000 random permutations of the data, and false detection rate of 0.05 used as a cut-off. Hiercarchical clustering [61] was performed in the D-Chip package using the correlation distance metric and average linkage [62]. For analysis using MapMan [29], mean log2 fold-change values for all 7,799 probe sets included in our original analysis were used for display and statistical testing using the Wilcoxon rank sum test. The Benjamini and Hochberg correction was applied to statistical tests in MapMan to take account of multiple hypothesis testing. Probe annotation and gene ontology (GO) term enrichment analysis were performed using the tools provided by the Tomato Functional Genomics Database [63]. P-values were corrected using the permutation algorithm within the analysis tool. Annotation files were the January 2010 versions.

### Gene expression analysis by RT-PCR

Following RNA extraction as outlined above and prior to cDNA synthesis, 10 μg RNA was treated with DNaseI (Invitrogen; http://www.invitrogen.com). cDNA was synthesized using SuperScript II reverse transcriptase (Invitrogen) using the primer GGCCACGCGTCGACTAGTAC(T)_16_VN. 30 cycles of PCR were carried out using Taq DNA polymerase (REDTaq; Sigma-Aldrich).

### Methyl viologen assay

Alethea pre-treatment was applied to tomato plants at 4.5 weeks, and wheat, maize, bean and brassica plants at 3.5 weeks (as detailed above) 24 h prior to the application of 500 μM methyl viologen (Sigma-Aldrich Ltd., Dorset, UK). MV was applied using a pressurized airbrush, and Silwet L-77 (De Sangosse Ltd., Cambridge, UK), a wetting agent, was added when applying the herbicide to maize and brassica plants to provide even application across the leaves. Control plants were treated with 0 μM MV (water) using the same method, and brassica and maize control plants received 0 μM MV (water) plus Silwet L-77 (0.025%). Once sprayed, plants were returned to the glasshouse and supplementary lighting switched off until the treatments had dried onto the leaves. 3 d after MV application, necrosis on the leaf surfaces was estimated visually as a percentage of the whole plant. Each species was subject to a minimum of two separate MV experiments, with the exception of tomato, which was assayed in four separate experiments.

## Competing interests

Transcriptomic analysis and photosynthetic performance assays were funded by Plant Impact PLC, the holders of related proprietary technology. While this manuscript is submitted with the agreement of Plant Impact PLC, this organisation is not funding the publication of this manuscript, and has not made an authorship contribution of any kind. The authors hold no financial investments in Plant Impact PLC, and do not hold any rights to any related patents or proprietary technologies.

## Authors’ contributions

JW co-designed and performed the experiments, co-analysed the data and co-wrote the manuscript, DP carried out the methyl-viologen sensitivity assay and analysed the data, NP conceived the study and co-designed the experiments, and MR designed and analysed the transcriptomics study and co-wrote the paper. All authors approved and have contributed to the final manuscript.

## Supplementary Material

Additional file 1 Details of the ‘Alethea’ composition. Breakdown of the components of the Alethea plant activator, including citation of relevant patent information as related to proprietary product technology. Click here for file

Additional file 2 Differentially-regulated gene lists derived from ‘Rank Products’ analysis of microarray data. Click here for file

Additional file 3 **Gene clusters derived from hierarchical cluster analysis. Full list of genes present in each of the clusters identified in Figure** 2**.** Data provided for each gene include Affymetrix probe set ID, the tomato UniGene ID corresponding to each probe ID and with TrEMBL database best sequence matches and corresponding E-values obtained from the Tomato Functional Genomics Database probe annotation service (http://ted.bti.cornell.edu/). Adjacent to each gene list are the outputs from GO term enrichment searches performed using the genes present within each cluster. Click here for file

Additional file 4 **Functional gene classes identified by MapMan as affected by Alethea and salinity.** Values show Benjamini & Hochberg-corrected P-values from the Wilcoxon rank sum test for MapMan gene classes (bins) that show significantly altered distributions of expression values (α = 0.05) for at least one relevant pair-wise comparison between groups identified using two-letter abbreviations, where the first letter indicates the pre-treatment (W; water, A; Alethea) and the second letter the main treatment (W; water, S; salt). Colours represent the general expression trend within the bin – tan; up-regulation, 10^-5^ < P < 0.05, red; up-regulation, p < 10^-5^, pale blue; down-regulation, 10^-5^ < P < 0.05, blue; down-regulation, p < 10^-5^. Click here for file

## References

[B1] Godfray HCJ Beddington JR Crute IR Haddad L Lawrence D Muir JF Pretty J Robinson S Thomas SM Toulmin C Food Security: the challenge of feeding 9 billion people Science 2010 327 5967 812 818 10.1126/science.1185383 20110467

[B2] Schmidhuber J Tubiello FN Global food security under climate change Proc Natl Acad Sci USA 2007 104 50 19703 19708 10.1073/pnas.0701976104 18077404 PMC2148361

[B3] Gregory PJ Johnson SN Newton AC Ingram JSI Integrating pests and pathogens into the climate change/food security debate J Exp Bot 2009 60 10 2827 2838 10.1093/jxb/erp080 19380424

[B4] Oerke EC Crop losses to pests J Agric Sci 2006 144 31 43 10.1017/S0021859605005708

[B5] Buchanan BB Gruissem W Jones RL Biochemistry & molecular biology of plants 2000 Rockville, Md.; [Great Britain]: American Society of Plant Physiologists

[B6] Varshney RK Bansal KC Aggarwal PK Datta SK Craufurd PQ Agricultural biotechnology for crop improvement in a variable climate: hope or hype? Trends Plant Sci 2011 16 7 363 371 10.1016/j.tplants.2011.03.004 21497543

[B7] Hu TT Kang SZ Li FS Zhang JH Effects of partial root-zone irrigation on hydraulic conductivity in the soil-root system of maize plants J Exp Bot 2011 62 12 4163 4172 10.1093/jxb/err110 21527627 PMC3153675

[B8] Wang YS Liu FL Andersen MN Jensen CR Improved plant nitrogen nutrition contributes to higher water use efficiency in tomatoes under alternate partial root-zone irrigation Funct Plant Biol 2010 37 2 175 182 10.1071/FP09181

[B9] Wargent JJ Elfadly EM Moore JP Paul ND Increased exposure to UV-B radiation during early development leads to enhanced photoprotection and improved long-term performance in *Lactuca sativa* Plant Cell Environ 2011 34 8 1401 1413 10.1111/j.1365-3040.2011.02342.x 21535014

[B10] Verhagen BWM Trotel-Aziz P Couderchet M Hofte M Aziz A Pseudomonas spp.-induced systemic resistance to *Botrytis cinerea* is associated with induction and priming of defence responses in grapevine J Exp Bot 2010 61 1 249 260 10.1093/jxb/erp295 19812243

[B11] Conrath U Beckers GJM Flors V Garcia-Agustin P Jakab G Mauch F Newman MA Pieterse CMJ Poinssot B Pozo MJ Priming: Getting ready for battle Mol Plant Microbe Interact 2006 19 10 1062 1071 10.1094/MPMI-19-1062 17022170

[B12] Macarisin D Wisniewski ME Bassett C Thannhauser TW Proteomic analysis of beta-aminobutyric acid priming and abscisic acid - induction of drought resistance in crabapple (*Malus pumila*): effect on general metabolism, the phenylpropanoid pathway and cell wall enzymes Plant Cell Environ 2009 32 11 1612 1631 10.1111/j.1365-3040.2009.02025.x

[B13] Tsai CH Singh P Chen CW Thomas J Weber J Mauch-Mani B Zimmerli L Priming for enhanced defence responses by specific inhibition of the Arabidopsis response to coronatine Plant J 2011 65 3 469 479 10.1111/j.1365-313X.2010.04436.x 21265899

[B14] Ashraf M Akram NA Arteca RN Foolad MR The physiological, biochemical and molecular roles of brassinosteroids and salicylic acid in plant processes and salt tolerance Crit Rev Plant Sci 2010 29 3 162 190 10.1080/07352689.2010.483580

[B15] Pauwels L Barbero GF Geerinck J Tilleman S Grunewald W Perez AC Chico JM Vanden Bossche R Sewell J Gil E NINJA connects the co-repressor TOPLESS to jasmonate signalling Nature 2010 464 7289 788 U169 10.1038/nature08854 20360743 PMC2849182

[B16] Ballare CL Jasmonate-induced defenses: a tale of intelligence, collaborators and rascals Trends Plant Sci 2011 16 5 249 257 10.1016/j.tplants.2010.12.001 21216178

[B17] Roberts MR Paul ND Seduced by the dark side: integrating molecular and ecological perspectives on the inflence of light on plant defence against pests and pathogens New Phytol 2006 170 4 677 699 10.1111/j.1469-8137.2006.01707.x 16684231

[B18] Zhu ZQ An FY Feng Y Li PP Xue L Mu A Jiang ZQ Kim JM To TK Li W Derepression of ethylene-stabilized transcription factors (EIN3/EIL1) mediates jasmonate and ethylene signaling synergy in Arabidopsis 2011 108 30 12539 12544 10.1073/pnas.1103959108 PMC3145709 21737749

[B19] Munns R Tester M Mechanisms of salinity tolerance Annu Rev Plant Biol 2008 59 651 681 10.1146/annurev.arplant.59.032607.092911 18444910

[B20] Moradi F Ismail AM Responses of photosynthesis, chlorophyll fluorescence and ROS-Scavenging systems to salt stress during seedling and reproductive stages in rice Ann Bot 2007 99 6 1161 1173 10.1093/aob/mcm052 17428832 PMC3243573

[B21] Fricke W Akhiyarova G Veselov D Kudoyarova G Rapid and tissue-specific changes in ABA and in growth rate in response to salinity in barley leaves J Exp Bot 2004 55 399 1115 1123 10.1093/jxb/erh117 15047763

[B22] Jakab G Ton J Flors V Zimmerli L Metraux JP Mauch-Mani B Enhancing Arabidopsis salt and drought stress tolerance by chemical priming for its abscisic acid responses Plant Physiol 2005 139 1 267 274 10.1104/pp.105.065698 16113213 PMC1203376

[B23] Luo ZB Janz D Jiang XN Gobel C Wildhagen H Tan YP Rennenberg H Feussner I Polle A Upgrading root physiology for stress tolerance by ectomycorrhizas: insights from metabolite and transcriptional profiling into reprogramming for stress anticipation Plant Physiol 2009 151 4 1902 1917 10.1104/pp.109.143735 19812185 PMC2785981

[B24] Tsonev TD Lazova GN Stoinova ZG Popova LP A possible role for jasmonic acid in adaptation of barley seedlings to salinity stress Journal of Plant Growth Regulation 1998 17 3 153 159 10.1007/PL00007029

[B25] James RA Rivelli AR Munns R von Caemmerer S Factors affecting CO_2_ assimilation, leaf injury and growth in salt-stressed durum wheat Funct Plant Biol 2002 29 12 1393 1403 10.1071/FP02069 32688739

[B26] Breitling R Armengaud P Amtmann A Herzyk P Rank products: a simple, yet powerful, new method to detect differentially regulated genes in replicated microarray experiments FEBS Lett 2004 573 1–3 83 92 15327980 10.1016/j.febslet.2004.07.055

[B27] Ouyang B Yang T Li HX Zhang L Zhang YY Zhang JH Fei ZJ Ye ZB Identification of early salt stress response genes in tomato root by suppression subtractive hybridization and microarray analysis J Exp Bot 2007 58 3 507 520 17210988 10.1093/jxb/erl258

[B28] Kiyosue T Yoshiba Y YamaguchiShinozaki K Shinozaki K A nuclear gene encoding mitochondrial proline dehydrogenase, an enzyme involved in proline metabolism, is upregulated by proline but downregulated by dehydration in Arabidopsis Plant Cell 1996 8 8 1323 1335 8776899 10.1105/tpc.8.8.1323 PMC161248

[B29] Thimm O Blasing O Gibon Y Nagel A Meyer S Kruger P Selbig J Muller LA Rhee SY Stitt M MAPMAN: a user-driven tool to display genomics data sets onto diagrams of metabolic pathways and other biological processes Plant J 2004 37 6 914 939 10.1111/j.1365-313X.2004.02016.x 14996223

[B30] Bukhov N Carpentier R Alternative Photosystem I-driven electron transport routes: mechanisms and functions Photosynth Res 2004 82 1 17 33 16228610 10.1023/B:PRES.0000040442.59311.72

[B31] Horváth EM Peter SO Joët T Rumeau D Cournac L Horváth GV Targeted Inactivation of the plastid ndhB gene in tobacco results in an enhanced sensitivity of photosynthesis to moderate stomatal closure Plant Physiol 2000 123 4 1337 1350 10.1104/pp.123.4.1337 10938352 PMC59092

[B32] Rumeau D Peltier G Cournac L Chlororespiration and cyclic electron flow around PSI during photosynthesis and plant stress response Plant Cell Environ 2007 30 9 1041 1051 10.1111/j.1365-3040.2007.01675.x 17661746

[B33] Dodge AD Some mechanisms of herbicide action Sci Prog 1975 62 247 447 466

[B34] Horváth E Szalai G Janda T Induction of abiotic stress tolerance by salicylic acid signaling Journal of Plant Growth Regulation 2007 26 3 290 300 10.1007/s00344-007-9017-4

[B35] Stevens J Senaratna T Sivasithamparam K Salicylic acid induces salinity tolerance in tomato (*Lycopersicon esculentum* cv. Roma): Associated changes in gas exchange, water relations and membrane stabilisation Plant Growth Regul 2006 49 1 77 83

[B36] del Amor FM Cuadra-Crespo P Alleviation of salinity stress in broccoli using foliar urea or methyl-jasmonate: analysis of growth, gas exchange, and isotope composition Plant Growth Regul 2011 63 1 55 62 10.1007/s10725-010-9511-8

[B37] Fedina IS Tsonev TD Effect of pretreatment with methyl jasmonate on the response of Pisum sativum to salt stress J Plant Physiol 1997 151 6 735 740 10.1016/S0176-1617(97)80071-5

[B38] Walia H Wilson C Condamine P Liu X Ismail AM Close TJ Large-scale expression profiling and physiological characterization of jasmonic acid-mediated adaptation of barley to salinity stress Plant Cell Environ 2007 30 4 410 421 10.1111/j.1365-3040.2006.01628.x 17324228

[B39] Dombrowski JE Salt stress activation of wound-related genes in tomato plants Plant Physiol 2003 132 4 2098 2107 10.1104/pp.102.019927 12913164 PMC181293

[B40] Capiati DA Pais SM Tellez-Inon MT Wounding increases salt tolerance in tomato plants: evidence on the participation of calmodulin-like activities in cross-tolerance signalling J Exp Bot 2006 57 10 2391 2400 10.1093/jxb/erj212 16766597

[B41] Cooper WR Jia L Goggin L Effects of jasmonate-induced defenses on root-knot nematode infection of resistant and susceptible tomato cultivars J Chem Ecol 2005 31 9 1953 1967 10.1007/s10886-005-6070-y 16132206

[B42] Thaler JS Jasmonate-inducible plant defences cause increased parasitism of herbivores Nature 1999 399 6737 686 688 10.1038/21420

[B43] Thaler JS Stout MJ Karban R Duffey SS Jasmonate-mediated induced plant resistance affects a community of herbivores Ecological Entomology 2001 26 3 312 324 10.1046/j.1365-2311.2001.00324.x

[B44] Beltrano J Ronco MG Montaldi ER Drought stress syndrome in wheat is provoked by ethylene evolution imbalance and reversed by rewatering, aminoethoxyvinylglycine, or sodium benzoate Journal of Plant Growth Regulation 1999 18 2 59 64 10.1007/PL00007049 10552133

[B45] Dat JF Lopez-Delgado H Foyer CH Scott IM Effects of salicylic acid on oxidative stress and thermotolerance in tobacco J Plant Physiol 2000 156 5–6 659 665

[B46] Kellos T Timar I Szilagyi V Szalai G Galiba G Kocsy G Stress hormones and abiotic stresses have different effects on antioxidants in maize lines with different sensitivity Plant Biology 2008 10 5 563 572 10.1111/j.1438-8677.2008.00071.x 18761495

[B47] Mur LAJ Kenton P Atzorn R Miersch O Wasternack C The outcomes of concentration-specific interactions between salicylate and jasmonate signaling include synergy, antagonism, and oxidative stress leading to cell death Plant Physiol 2006 140 1 249 262 16377744 10.1104/pp.105.072348 PMC1326048

[B48] Uppalapati SR Ayoubi P Weng H Palmer DA Mitchell RE Jones W Bender CL The phytotoxin coronatine and methyl jasmonate impact multiple phytohormone pathways in tomato Plant J 2005 42 201 217 10.1111/j.1365-313X.2005.02366.x 15807783

[B49] Cao WH Liu J He XJ Mu RL Zhou HL Chen SY Zhang JS Modulation of ethylene responses affects plant salt-stress responses Plant Physiol 2007 143 2 707 719 17189334 10.1104/pp.106.094292 PMC1803741

[B50] Shi HZ Kim Y Guo Y Stevenson B Zhu JK The Arabidopsis *SOS5* locus encodes a putative cell surface adhesion protein and is required for normal cell expansion Plant Cell 2003 15 19 32 10.1105/tpc.007872 12509519 PMC143448

[B51] Walia H Wilson C Condamine P Liu X Ismail AM Zeng LH Comparative transcriptional profiling of two contrasting rice genotypes under salinity stress during the vegetative growth stage Plant Physiol 2005 139 822 835 10.1104/pp.105.065961 16183841 PMC1255998

[B52] He Y Zhu ZJ Exogenous salicylic acid alleviates NaCl toxicity and increases antioxidative enzyme activity in Lycopersicon esculentum Biologia Plantarum 2008 52 4 792 795 10.1007/s10535-008-0155-8

[B53] Sasaki-Sekimoto Y Taki N Obayashi T Aono M Matsumoto F Sakurai N Suzuki H Hirai MY Noji M Saito K Coordinated activation of metabolic pathways for antioxidants and defence compounds by jasmonates and their roles in stress tolerance in Arabidopsis Plant J 2005 44 4 653 668 10.1111/j.1365-313X.2005.02560.x 16262714

[B54] Shan CJ Liang ZS Jasmonic acid regulates ascorbate and glutathione metabolism in Agropyron cristatum leaves under water stress Plant Sci 2010 178 2 130 139 10.1016/j.plantsci.2009.11.002

[B55] Zhang XH Shen L Li FJ Zhang YX Meng DM Sheng JP Up-regulating arginase contributes to amelioration of chilling stress and the antioxidant system in cherry tomato fruits J Sci Food Agric 2010 90 13 2195 2202 10.1002/jsfa.4070 20628998

[B56] Groppa MD Benavides MP Polyamines and abiotic stress: Recent advances Amino Acids 2008 34 1 35 45 10.1007/s00726-007-0501-8 17356805

[B57] Chen H McCaig BC Melotto M He SY Howe GA Regulation of plant arginase by wounding, jasmonate, and the phytotoxin coronatine J Biol Chem 2004 279 44 45998 46007 10.1074/jbc.M407151200 15322128

[B58] Verwoerd TC Dekker BMM Hoekema A A small-scale procedure for the rapid isolation of plant RNAs Nucleic Acids Res 1989 17 6 2362 10.1093/nar/17.6.2362 2468132 PMC317610

[B59] Wu ZJ Irizarry RA Gentleman R Martinez-Murillo F Spencer F A model-based background adjustment for oligonucleotide expression arrays JAmStatistical Association 2004 99 468 909 917

[B60] Saeed AI Bhagabati NK Braisted JC Liang W Sharov V Howe EA Li J Thiagarajan M White JA Quackenbush J TM4 microarray software suite Methods Enzymol 2006 411 134 193 16939790 10.1016/S0076-6879(06)11009-5

[B61] Eisen MB Spellman PT Brown PO Botstein D Cluster analysis and display of genome-wide expression patterns Proceedings of the National Academy of Sciences of the United States of America 1998 95 25 14863 14868 10.1073/pnas.95.25.14863 9843981 PMC24541

[B62] Parmigiani G The analysis of gene expression data methods and software 2003 New York; London: Springer

[B63] Fei Z Joung JG Tang X Zheng Y Huang M Lee JM McQuinn R Tieman DM Alba R Klee HJ Giovannoni JJ Tomato Functional Genomics Database: a comprehensive resource and analysis package for tomato functional genomics Nucleic Acids Res 2011 39 1156 1163 10.1093/nar/gkq991 PMC3013811 20965973

