# Supplementary material for: Reduction of photosynthetic sensitivity in response to abiotic stress in tomato is mediated by a new generation plant activator
Source: BMC Plant Biol. 2013 Jul 30;13:108. doi: 10.1186/1471-2229-13-108 (PMC3733976; doi:10.1186/1471-2229-13-108)
Supplement: Additional file 1 — Details of the ‘Alethea’ composition. Breakdown of the components of the Alethea plant activator, including citation of relevant patent information as related to proprietary product technology. [file 1471-2229-13-108-S1.pdf]

Additional file 1

The 'Alethea' plant activator was supplied by Plant Impact PLC (Preston, UK), and was composed of a combination of potassium dihydrojasmonate (PDJ), sodium benzoate (SB), and L-arginine (Arg), as defined by the family of international patents arising from PCT/GB2005/001562. The composition was further augmented by several plant nutrients (Table 1).

Table 1. Nutritional constituents of this experimental formulation of the Alethea formula. All values quoted are prior to dilution of Alethea compound for experimental use.

Nutrient	% contribution to Alethea compound
Zinc (Zn)	2.0
Iron (Fe)	2.0
Manganese (Mn)	1.0
Copper (Cu)	0.5
Magnesium oxide (MgO)	0.5
Boron (B)	0.025
